# Standardization and Validation of Fluorescence-Based Quantitative Assay to Study Human Platelet Adhesion to Extracellular-Matrix in a 384-Well Plate

**DOI:** 10.3390/ijms21186539

**Published:** 2020-09-07

**Authors:** Augusto Martins Lima, Damian S. Saint Auguste, François Cuenot, Ana C. Martins Cavaco, Tom Lachkar, Cindy Marie Elodie Khawand, Rodrigo A. Fraga-Silva, Nikolaos Stergiopulos

**Affiliations:** 1 Laboratory of Hemodynamics and Cardiovascular Technology (LHTC), Institute of Bioengineering, École Polytechnique Fédérale de Lausanne, 1015 Lausanne, Switzerland; damian.stauguste@gmail.com (D.S.S.A.); francois.cuenot@epfl.ch (F.C.); tom.lachkar@epfl.ch (T.L.); khawand.cindy@gmail.com (C.M.E.K.); rodrigo.fragasilva@epfl.ch (R.A.F.-S.); nikolaos.stergiopulos@epfl.ch (N.S.); 2 Institute of Bioengineering, École Polytechnique Fédérale de Lausanne Station 09, MED 3.2924, CH-1015 Lausanne, Switzerland; 3 Laboratory for Orthopaedic Technology, Institute for Biomechanics, Swiss Federal Institute of Technology Zurich, 8093 Zurich, Switzerland; 4 Luis Costa Lab, Instituto de Medicina Molecular, Faculdade de Medicina da Universidade de Lisboa, 1649-028 Lisboa, Portugal; anacavaco@medicina.ulisboa.pt

**Keywords:** platelet adhesion, extracellular matrix, fluorescence-based quantitative assay, BCECF-AM, 384-well plate, high-throughput screening assay

## Abstract

Platelets play a crucial role in the immunological response and are involved in the pathological settings of vascular diseases, and their adhesion to the extracellular matrix is important to bring leukocytes close to the endothelial cells and to form and stabilize the thrombus. Currently there are several methods to study platelet adhesion; however, the optimal parameters to perform the assay vary among studies, which hinders their comparison and reproducibility. Here, a standardization and validation of a fluorescence-based quantitative adhesion assay to study platelet-ECM interaction in a high-throughput screening format is proposed. Our study confirms that fluorescence-based quantitative assays can be effectively used to detect platelet adhesion, in which BCECF-AM presents the highest sensitivity in comparison to other dyes.

## 1. Introduction

Platelet adhesion is a crucial step in bleeding control and in the thrombosis process. Although circulating platelets show no interaction between them, neither with the internal surface of normal vessels, platelets promptly adhere to exposed extracellular matrix (ECM) proteins upon vascular injury or in pathological settings [1]. Therefore, the balance between hemostasis and thrombosis depends on platelet-ECM interaction: inefficient adhesion leads to bleeding, whereas excessive adhesion followed by platelet activation can prompt thrombus formation [2]. Upon endothelial cell damage, depending on the depth of the injury, platelets come into contact with different ECM constituents [2]. Interestingly, platelets can also adhere to damaged endothelial cells [3], although ECM is their main adhesive substrate. Interaction with ECM leads to platelet rapid activation and prothrombotic factors release, which recruit additional platelets from the bloodstream and promotes platelet aggregation. Platelet adhesion is an initial and crucial step of the coagulation cascade which prevents posttraumatic blood loss [4]. However, uncontrolled thrombus formation can lead to vascular occlusion, ischemia, and if located in coronary and cerebral arteries, myocardial infarction and ischemic stroke, respectively [5,6,7]. Furthermore, platelet adhesion might also influence atherosclerotic plaque progression and stability during the development of atherosclerosis [6].

Vessels are lined by endothelium, and beneath this layer rests the basement membrane, which contains type IV collagen, laminins, nidogens, and perlecan [8]. While the predominant isoforms in vascular basement membranes are nidogen 2 and laminins-411 and -511, in the interstitium surrounding the vessels, fibrillar collagen types I, III, and V are the most prominent [9]. Other adhesive proteins, such as von Willebrand factor (vWF), fibronectin, vitronectin, and fibrinogen, are present in plasma and in platelet α-granules [10]. For instance, fibrinogen and vitronectin although not synthesized by vascular cells, are potentially relevant thrombogenic substrates as they immobilize onto ECM at sites of injury, being incorporated in the thrombus [11]. Thrombogenic substrates encountered by platelets are determined by the injury degree and depend on the vessel type, influencing platelet adhesion efficiency [10]. Damage to the endothelial layer exposes type IV collagen, laminins, vWF, and fibronectins, whereas more extensive injuries reaching the smooth muscle layer or interstitial ECM expose fibrillar collagens, elastin and microfibrils [2,12]. Furthermore, platelets adhesion capacity to ECM proteins varies, which might also influence the adhesion strength and efficiency [10].

Platelet adhesion to ECM proteins is facilitated by the synergistic function of several platelet receptors, which are essential for the initial adhesion and the subsequent platelet activation and aggregation [6]. The main signals triggered during platelet activation in response to ECM proteins arise from receptors such as glycoprotein (GP) VI, together with integrin α2β1, both mainly binding to collagen [6,13]. Full platelet activation is further facilitated by the transient interaction between platelet-specific GPIbα and vWF [6,13]. Following platelet adhesion and activation, aggregation is enabled by integrin αIIbβ3, which changes its ligand binding properties and acquires the ability to bind soluble adhesive proteins, including fibrinogen and vWF [6]. Gene-targeted mouse models were crucial in understanding the relative function of the different platelet receptors. It confirms the importance of adhesion receptors such as GPIbα or integrin αIIbβ3 in thrombus formation, whereas the function of receptors, such as GPVI, seems to be regulated by the severity of injury, type of vessel injured, and generated signaling pathways [14]. Overall the thrombus is stabilized by platelets interacting with collagen present in the vessel ECM during adhesion, and by fibrinogen/fibrin bound-activated platelets during aggregation [6].

Vascular diseases are some of the major clinical problems in the developed world [5,15], and given the relevance of platelets in these pathologies, the understanding of platelets adhesion to the ECM is an important topic of research. Furthermore, new evidences highlight platelet adhesive properties as central to a variety of pathophysiological processes such as inflammation [16], immune-mediated host defense [17], and cancer metastasis [18].

Several methodologies to measure platelet adhesion have been described [4,19,20]; however, there is no standard test, which impairs comparison of results from different studies, contributing to the lack of reproducibility in science. Increasing recognition of the inability to replicate the majority of fundamental, biomedical, and preclinical research findings presented in high-profile journals, emphasizes the problem of irreproducibility [21]. Therefore, in the present work a standardization and validation of a fluorescence-based quantitative adhesion assay to study platelet-ECM interaction using a 384-well plate is proposed.

## 2. Results

### 2.1. Assay Optimization

#### 2.1.1. Assay Linearity

**Figure 1:**
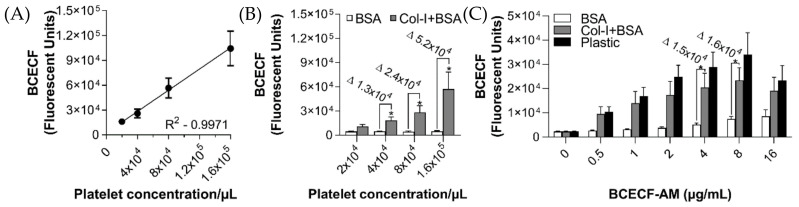
Platelet adhesion assay linearity in a 96-well plate. 96-well microplates were coated with collagen-I (8 µg/mL in 50 µL) or incubated with distillated water for 1 h at 37 °C. After blocking the wells with BSA (0.03%), different concentrations of human washed platelets (2 × 10^4^ to 1.6 × 10^5^/µL in 100µL) were added, followed by incubation for 1 h at 37 °C. Next, non-adherent platelets were removed, and adherent platelets were incubated with BCECF-AM (0.25 to 16 µg/mL in 50 µL) for 30 min at 37 °C. Fluorescence intensity was measured using a plate reader (VictorX, PerkinElmer, Waltham, MA, USA). (**A**) Assay linearity (R^2^ = 0.9971) was calculated by measuring the fluorescence signal of different platelet concentrations adhered to plastic. (**B**) The optimum platelet concentration to obtain statistical differences between non-coated (white bars) and collagen-I-coated wells (grey bars) was assessed by using different platelet concentrations. Δ represents delta: (Col-I+BSA) − (BSA). (**C**) The optimal concentration of BCECF-AM was assessed by comparing the fluorescence signal of adherent platelets (8 × 10^4^/µL in 100µL) on non-coated wells (white bars) and collagen-I-coated wells (grey bars). Plastic was used as positive control (black bars). Δ represents delta (Col-I+BSA) − (BSA). Fluorescence intensity of collagen-coated surfaces blocked with BSA was compared to BSA alone for each experimental group by *t*-test (* *p* < 0.05, data are mean ± SEM; *n* = 4).

Assay linearity was calculated by measuring the adhesion to plastic of different concentrations of BCECF-stained human washed platelets. In the proposed assay, the fluorescence signal increased in a platelet concentration-dependent manner (fluorescent units: 2 × 10^4^ = 16,013 ± 2,736; 4 × 10^4^ = 26,115 ± 5,442; 8 × 10^4^ = 56,809 ± 12,000; 1.6 × 10^5^ = 104,408 ± 20,797—R^2^ = 0.9971, Figure 1A). In addition, 4 × 10^4^/µL was the minimal platelet concentration necessary to detect significant changes in platelet adhesion to collagen-I (fluorescent units: BSA = 4671 ± 627 vs. Col-I = 18,175 ± 4642) and no statistical differences were found among the groups with platelet concentration of 2 × 10^4^/µL (fluorescent units: BSA = 4588 ± 636 vs. Col-I = 10,667 ± 5213, Figure 1B). The optimal concentration of BCECF-AM was also tested by measuring the platelet adhesion to collagen-I. As shown in the Figure 1C, the fluorescence signal was significantly increased on adherent platelets stained with 4 µg/mL (fluorescent units: BSA = 5146 ± 669 vs. Col-I = 20,464 ± 5817) and 8 µg/mL (fluorescent units: BSA = 7472 ± 1064 vs. Col-I = 23,361 ± 5291) of BCECF-AM. Interestingly, at high concentrations of BCECF-AM (16 µg/mL), there was no statistical difference between platelet adhesion to collagen-I compared to non-coated wells (fluorescent units: BSA = 8590 ± 2653 vs. Col-I = 19,069 ± 5560). These results demonstrate the feasibility of platelet adhesion measurement using BCECF-AM and confirm that platelet and BCECF-AM concentrations are important parameters to detect statistical differences between platelet adhesion to ECM-coated and non-coated wells.

#### 2.1.2. Non-Specific Binding Blocking

**Figure 2:**
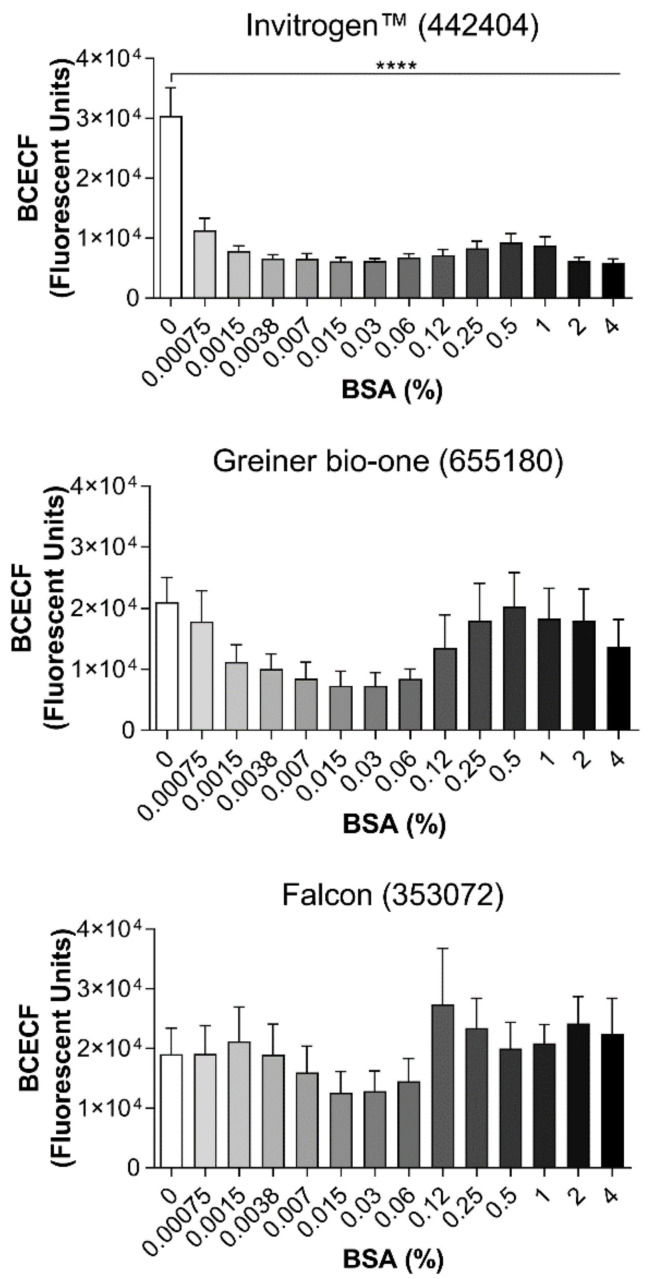
BSA blocking on different types of 96-well plates. BSA was used to prevent non-specific adhesion of platelets to the plastic. To find the optimum conditions, 3 types of 96-well plates (Invitrogen™—442404; Greiner Bio-one—655,180 and Falcon—353072) were blocked with different concentrations of BSA (0.00075 to 4% in 50 µL) for 1 hour at 37 °C. Next, human washed platelets (8 × 10^4^/µL in 100 µL) were added, followed by incubation for 1 h at 37 °C. Non-adherent platelets were removed, and adherent platelets were incubated with BCECF-AM (4 µg/mL in 50 µL) for 30 min at 37 °C. Fluorescence intensity was measured using a plate reader (VictorX, PerkinElmer, Waltham, MA, USA). Values were compared to the control condition—without BSA—by one-way ANOVA followed by Dunnett’s post hoc test (**** *p* < 0.0001, data are mean ± SEM; *n* = 4).

BSA has been widely used to prevent the non-specific binding of cells to different surfaces, including polystyrene, assuring the specificity of cell adhesion to the ECM. However, the BSA concentration varies largely between experimental protocols, as well as the type of plate used. Therefore, we tested the optimal BSA concentration to prevent non-specific binding using 3 different microplates (Figure 2). In this experimental setup, the following microplates were used: (1) Invitrogen™ (44-2404-21) has the MaxiSorp^®^ technology with a highly charged polystyrene surface with high affinity for molecules with polar or hydrophilic groups; (2) Greiner Bio-one (655180) and (3) Corning (353072) have polystyrene surface wells treated with plasma gas, which increases hydrophobicity. To block non-specific platelet binding to the wells surface, different BSA concentrations were used (0.00075 to 4%—BSA) and all concentrations reduced significantly the platelet adhesion to the plastic of the Invitrogen™ (44-2404-21) plate with the MaxiSorp^®^ technology. Furthermore, 0.03% BSA had the best blocking effect compared to all other BSA concentrations (fluorescent units: 0% BSA = 30,404 ± 4713 vs. 0.03% BSA = 6184 ± 400). Surprisingly, although there was a decreasing tendency of platelet adhesion to plastic at 0.03% BSA concentration, no statistical difference was found in neither BSA concentrations used to block Greiner Bio-one (655180) or Corning (353072) plates. These results clearly show that BSA low concentrations inhibit platelet adhesion to plastic of Invitrogen™ (44-2404-21) plate with the MaxiSorp^®^ technology.

#### 2.1.3. Optimal ECM Protein Concentration to Perform Platelet Adhesion Assay

**Figure 3:**
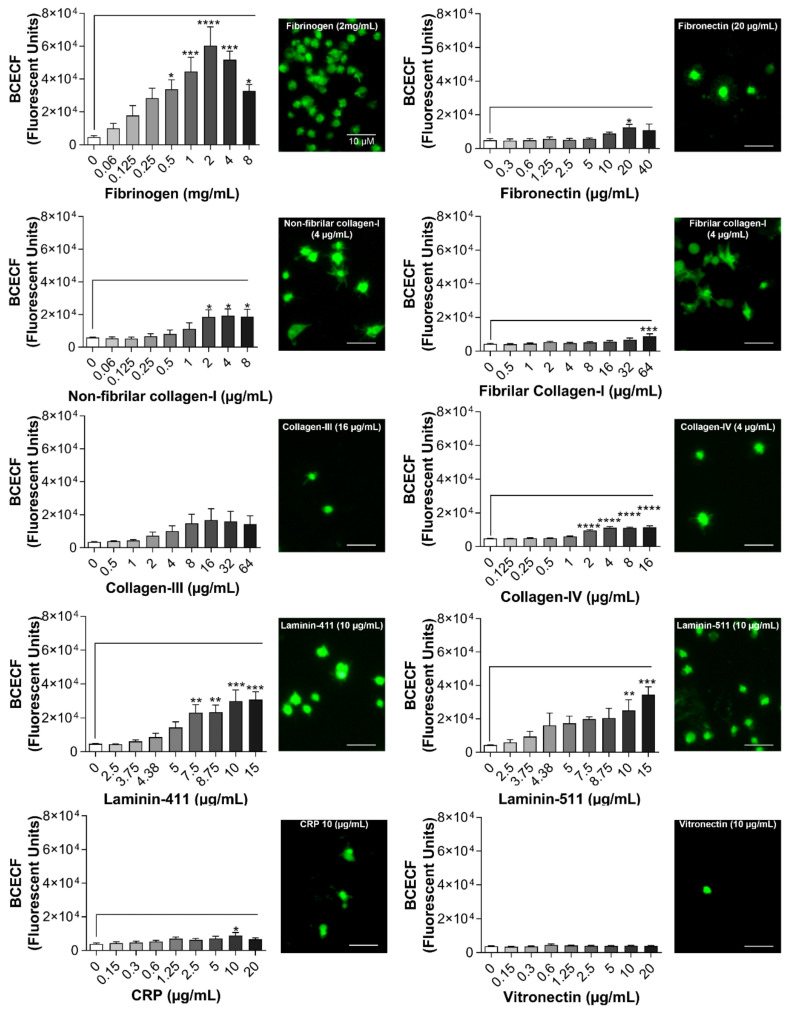
Platelet adhesion on different ECM proteins measured by BCECF-AM. Coating of 96-well microplates with ECM proteins (50 µL) was performed for 1 h at 37 °C. Different proteins and concentrations of ECM were used: fibrinogen (0.06 to 8 mg/mL); fibronectin (0.3 to 40 µg/mL); non-fibrillar collagen-I (0.06 to 8 µg/mL); fibrillar collagen-I (0.06 to 8 µg/mL); collagen-III (0.5 to 64 µg/mL); collagen-IV (0.125 to 16 µg/mL); laminin-411 (2.5 to 15 µg/mL); laminin-511 (2.5 to 15 µg/mL); collagen-related peptide (CRP) (0.15 to 20 µg/mL); vitronectin (0.15 to 20 µg/mL). In the wells without coating distillated water was added during the coating incubation time. After blocking the wells with BSA (0.03%), platelets (8 × 10^4^/µL in 100µL) were added, followed by incubation for 1 h at 37 °C. Next, non-adherent platelets were removed, and adherent platelets were incubated with BCECF-AM (4 µg/mL in 50 µL) for 30 min at 37 °C. Fluorescence intensity was measured using a plate reader (VictorX, PerkinElmer, Waltham, MA, USA) and images were taken using a fluorescence microscopy (Eclipse Ti2, Nikon, Tokyo, Japan) with a 20× objective (scale bar 10 µM). Values were compared with the non-coated control condition by one-way ANOVA followed by Dunnett’s post hoc test (* *p* < 0.05, ** *p* < 0.01, *** *p* < 0.001, **** *p* < 0.0001, data are mean ± SEM; *n* = 4).

Platelet adhesion is an important function in response to vascular damage and several ECM proteins are involved in this event, depending on the type and depth of the injury. In order to test the BCECF-AM-based assay capability to measure platelet adhesion, several ECM proteins were tested at different concentrations: fibrinogen, fibronectin, non-fibrillar and fibrillar collagen-I, collagen-III, collagen-IV, laminin-411, laminin-511, CRP, and vitronectin (Figure 3). Platelet adhesion was significantly increased on wells coated with fibrinogen, followed by laminin-511, laminin-411, non-fibrillar collagen-I and collagen-IV, in a concentration-dependent manner. Interestingly, at high concentrations, platelet adhesion to fibrinogen was markedly impaired. Platelet adhesion was also detected on fibronectin, fibrillar collagen-I and CRP, only at one specific concentration (fibronectin: 20 µg/mL; fibrillar collagen-I: 64 µg/mL and CRP: 10 µg/mL). No statistical difference on platelet adhesion was detected on wells coated with collagen-III or vitronectin. Altogether these results show the BCEFC-AM-based assay capability to detect human platelet adhesion to the most relevant ECM proteins in vessel damage and thrombus formation.

### 2.2. Assay Validation

#### 2.2.1. Comparison between BCECF-AM vs. Calcein-AM and Its Variations

**Figure 4:**
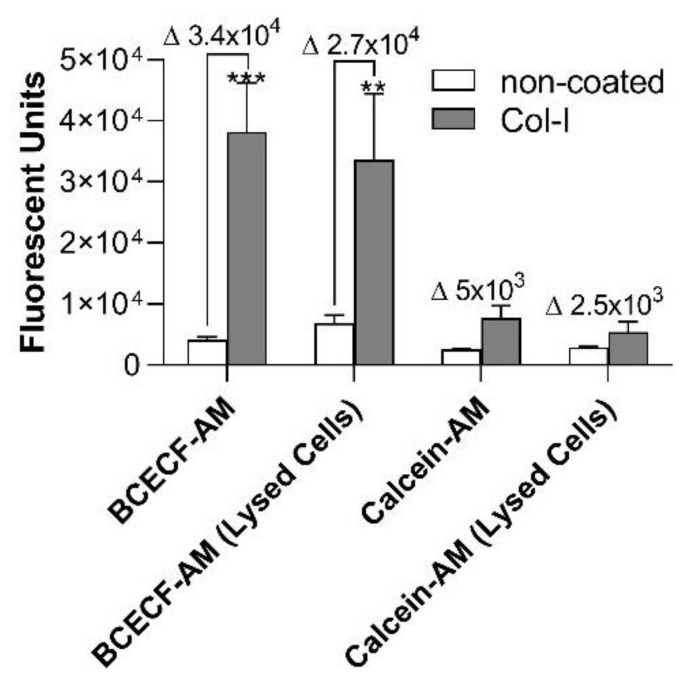
Platelet adhesion detection using different techniques: BCECF-AM vs. Calcein-AM. Coating of 96-well microplates with collagen-I (4 µg/mL in 50 µL) or distillated water were performed for 1 h at 37 °C. After blocking the wells with BSA (0.03%), human washed platelets (8 × 10^4^/µL in 100 µL) were added, followed by incubation for 1 h at 37 °C. Next, non-adherent platelets were removed. For the BCECF-AM experimental group, adherent platelets were incubated with BCECF-AM (4 µg/mL in 50 µL) for 30 min at 37 °C. After washing the excess of BCECF-AM, fluorescence intensity was measured using plate reader (VictorX, PerkinElmer, Waltham, MA, USA). In an independent experimental condition, the signal of BCECF-AM was measured after lysing the adhered platelets with lysis buffer. For the experiment using Calcein, AM (2 µg/mL), platelets were pre-stained following the protocol previous described by several authors (see Table 1). Briefly, PRP obtained by centrifugation of the whole blood was incubated with Calcein-AM (2 µg/mL) for 1 h at 37 °C. Platelets were then washed as described in the Section 4.2. Fluorescence intensity was measured using plate reader (VictorX, PerkinElmer, Waltham, MA, USA). Δ represents delta: (Col-I) − (non-coated). Values were compared with the control condition with BSA only (non-coated), by two-way ANOVA followed by Tukey’s post hoc test (** *p* < 0.01, *** *p* < 0.001, data are mean ± SEM; *n* = 4).

In order to validate the assay, BCECF-AM-based assay was compared to two other labelling techniques: Calcein-AM (Figure 4) and Sudan Black B (SBB (Supplementary Figure S1). Platelets were pre-stained as previously described by several authors, using Calcein-AM (see Table 1) or SBB [19]. In contrast, the experimental protocol employing BCECF-AM stains the already adherent platelets. For this experimental setup, all techniques were performed with the same platelet, collagen-I and BSA concentrations. In addition, two different approaches were used to detect the platelet adhesion signal: Non-lysed vs. lysed platelets. Surprisingly, only the BCECF-AM technique was capable to detect platelet adhesion with statistical differences between non-coated vs. collagen-I-coated wells (fluorescent units: non-coated wells = 4114 ± 537 vs. 4 µg/mL collagen-I-coated wells = 38,114 ± 8061), while for Calcein-AM (Figure 4) no statistical differences were observed between both conditions. In addition, a more robust delta value of the fluorescence signal for the measurements was observed for the non-lysed BCECF-AM labeled platelets. This experiment clearly shows that BCECF-AM is a more accurate technique to measure platelet adhesion compared to Calcein-AM. Interestingly, although a significant difference between non-coated vs. collagen-coated wells was observed when platelets were stained with SBB, platelet aggregates were detected on non-coated wells (supplementary Figure S1).

**Figure 5:**
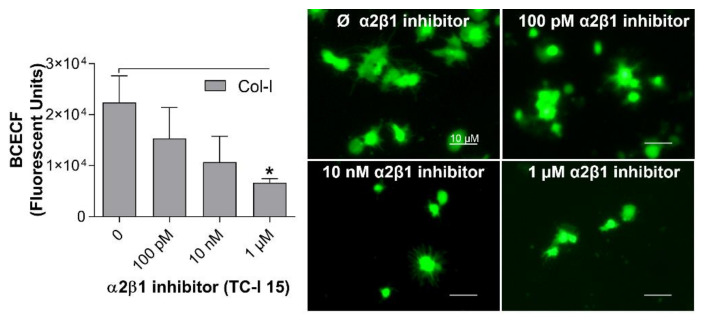
Sensitivity of the optimized assay to detect platelet adhesion inhibition. Coating of 96-well microplates with collagen-I (4 µg/mL in 50 µL) or distillated water was performed for 1 h at 37 °C. After blocking the wells with BSA (0.03%), human washed platelets (8 × 10^4^/µL in 100µL) containing different concentrations of TC-I 15, an α2β1 integrin inhibitor, were added to the coated wells, followed by incubation for 1 h at 37 °C. Non-adherent platelets were removed, and adherent platelets were incubated with BCECF-AM (4 µg/mL in 50 µL) for 30 min at 37 °C. Fluorescence intensity was measured using a plate reader (VictorX, PerkinElmer). Values were compared to the control group without TC-I 15 by one-way ANOVA followed by Dunnett’s post hoc test (* *p* < 0.05, data are mean ± SEM; *n* = 4). Images were acquired with a fluorescence microscopy (Eclipse Ti2, Nikon, Waltham, MA, USA) with a 20x objective (scale bar 10 µM).

TC-I 15, a potent α2β1 integrin inhibitor, was used to test the assay’s ability to test different platelet receptor inhibitors and their effect on platelet adhesion. As shown in Figure 5, platelet adhesion on collagen-I-coated wells was markedly reduced by TC-I 15 in a concentration-dependent manner. TC-I 15, at a concentration of 1µM, inhibited significantly platelet adhesion to collagen-I (fluorescent units: non-treated platelets = 22,390 ± 5277 vs. TC-I 15 treated platelets, 1 µM = 6633 ± 815) and the dendritic shape with spiky membrane extensions, typically caused by adhesion to collagen-I, was abolished. Taken together, these results demonstrate the relevance of the assay in detecting platelet adhesion inhibition in pharmacological tests.

#### 2.2.2. Z′-Factor Calculation in a 384-Well Plate

**Figure 6:**
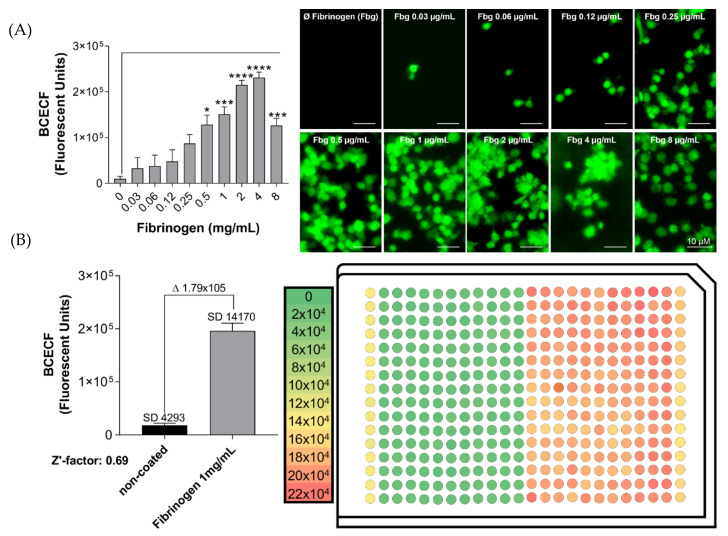
Z′-factor calculation to validate the platelet adhesion assay in a 384-well microplate. (**A**) Platelet adhesion similarity between 96- and 384-well microplates. Coating of 384-well microplate with different concentrations of fibrinogen (Fbg 0.03 to 8 mg/mL in 20 µL), or incubation with distillated water, were performed for 1 h at 37 °C. After blocking the wells with BSA (0.03%), human washed platelets (8 × 10^4^/µL in 10µL) were added, followed by incubation for 1 h at 37 °C. Next, non-adherent platelets were removed, and adherent platelets were incubated with BCECF-AM (4 µg/mL in 20 µL) for 30 min at 37 °C. Fluorescence intensity was measured using a plate reader (VictorX, PerkinElmer, Waltham, MA, USA). Values were compared with the control condition (non-coated plastic) by one-way ANOVA followed by Dunnett’s post hoc test (* *p* < 0.05, *** *p* < 0.001, **** *p* < 0.000,1 values presented as SEM resulting from duplicate average of four independent experiments). Images were acquired using a fluorescence microscopy (Eclipse Ti2, Nikon) with a 20x objective. (**B**) Z′-factor calculation to validate the platelet adhesion assay. In a 384-well microplate, 20 µL of water were added to half of the microplate (176 wells—left side). In the other half, fibrinogen (1 mg/mL) diluted in water was added (176 wells—right side). After blocking the wells with BSA (0.03%), human washed platelets (8 × 10^4^/µL in 10 µL) were added, followed by incubation for 1 h at 37 °C. Next, non-adherent platelets were removed, and adherent platelets were incubated with BCECF-AM (4 µg/mL in 20 µL) for 30 min at 37 °C. As a control, platelet adhesion on plastic was measured in the first and last columns. Δ represents delta: (Fibrinogen) − (non-coated). Fluorescence intensity (F.I.) was measured using a plate reader (VictorX, PerkinElmer, Waltham, MA, USA). The data of the graphs showing the Z′-factor were calculated using the equation previously published by Zhang et al. [22].

Platelet adhesion to fibrinogen was studied to validate the assay in a 384-well plate. The adhesion response was compared to the results obtained using a 96-well plate. The same fibrinogen, BSA and platelet concentration was used in both plates with a lower final volume for 384-well plate compared to 96-well plate (see Standard Operating Procedure supplemental material). As it is shown on Figure 6, platelet adhesion to fibrinogen on 384-well plate increases in a concentration-dependent manner. The maximal response was obtained at 4 mg/mL of fibrinogen, and the response was significantly increased compared to BSA coated-wells (concentrations 0.25 mg/mL to 8 mg/mL) (Figure 6A). Similarly to the 96-well plate, platelet adhesion was impaired when high concentration of fibrinogen was used (8 mg/mL). Although the maximal response varied between both plates (fluorescent units: 384-well plate, 4 mg/mL fibrinogen = 231,032 ± 12,022 vs. 96-well plate, 2 mg/mL fibrinogen = 60,411 ± 11326), the pattern of platelet adhesion response was clearly similar, validating the assay also to a 384-well plate. 

The Z′-factor, a statistical parameter that considers the window and variance around high and low assay signals, was used to measure the quality of the assay. Importantly, the Z′-factor ranges −∞ to 1 and an assay with a score greater than 0.5 is considered robust and appropriate to high-throughput screening [22]. To examine this, fibrinogen (1 mg/mL) diluted in water was added to half of the 384-well microplate (176 wells—right side). In the other half, water was added (176 wells—left side). After blocking the wells with BSA, platelet adhesion was measured using BCECF-AM. As shown on Figure 6B, using a platelet concentration of 8 × 10^4^/µL, an optimal Z′-factor value (<0.5) was attained with a 384-well plate (fluorescent units: non-coated wells = mean 17,819 ± SD 4293 vs. fibrinogen-coated wells, 1 mg/mL = mean 196,492 ± SD 14170).

## 3. Discussion

Adhesion to different substrates is an important step in physiological and pathological processes involving platelets. Events such as thrombosis depend on platelet adhesion to the exposed vessel ECM. Methods assessing platelet adhesion, allow the study of platelet’s capacity to adhere to different ECM proteins, and also investigate adhesion inhibitors, pathophysiological processes as inflammation, immune-mediated host defense, and cancer metastasis. Several methods have been described and widely used, employing different platelet adhesion detection methods. In the present study, we selected two methods to further characterize and compare, employing either BCECF-AM or Calcein-AM. Our work, by standardizing the method, is relevant mainly in the contexts of scientific reproducibility and decision making, regarding the selection of submaximal concentrations of adhesion inhibitors.

The selected cell-permeable dyes to be investigated in the present work, BCECF-AM and Calcein-AM, were previously used with similar purposes and the original articles employing these methods were gathered in Table 1 and Table 2, respectively. Although experiments employing SBB were also performed, our experiments hinted that this dye is not suitable to stain platelets, since it leads to platelet aggregation, when no substrate was added to the wells (non-coated conditions).

**Table 1:** Previous studies using Calcein-AM to measure platelet adhesion.

Year	Type of Plate	ECM Concentration	Blocking BSA	Platelet Concentration	Dye Concentration	Type of Measurement	Ref.
2000	MicroFLUOR 96-well (Dynatech)	Collagen (2 μg/well) or CRP (1.5 μg/well)	5%	N/A	Pre-labeled platelets with 2 μM Calcein-AM at RT, 30 min	Plate reader without lysing platelets	[23]
2000	Microfluor 96-well (Dynatech)	Collagen (2 μg/well) or Convulxin (1.5 μg/well)	N/A	N/A	Pre-labeled platelets Calcein-AM	Plate reader without lysing platelets	[24]
2002	96-well (N/A)	Vitronectin (5 μg/mL) or Fibrinogen (10 μg/mL)	3%	100,000/μL	Pre-labeled platelets with 2.5 μM Calcein-AM at RT, 30 min	Plate reader after lysing platelets	[25]
2003	Tissue culture 96-well (Costar)	D100 or D98 Fibrinogen fragments (20 μg/mL)	1%	100,000/μL	Pre-labeled platelets with 10 μM Calcein-AM at 37 °C, 30 min	Plate reader without lysing platelets	[26]
2004	MaxiSorp 96-well (Invitrogen™)	N/A	2%	200,000/μL	Pre-labeled platelets withn 2 μM Calcein-AM at 37 °C, 60 min	Microscopy	[27]
2006	96-well (Greiner)	Fibronectin (5 μg/well)	N/A	N/A	Pre-labeled platelets with 5 μM Calcein-AM, 60 min	Plate reader without lysing platelets	[28]
2006	96-well (N/A)	Fibronectin (5 µg/well) or Collagen-III (1 µg/well)	N/A	1000/μL	Pre-labeled platelets with 5 μM Calcein-AM, 60 min	Plate reader without lysing platelets	[29]
2008	384-well (Corning no. 3711)	Fibrinogen (50 μg/mL)	0.35%	250,000/μL	Pre-labeled platelets with 7 μM Calcein-AM at RT, 30 min	Plate reader without lysing platelets	[30]
2010	Immulon 4HBX 96-well (ThermoLabsystems)	Fibrinogen (0.1 to 50 μg/mL)	1%	100,000/μL	Pre-labeled platelets with 10 μM Calcein-AM at 37 °C, 30 min	Plate reader without lysing platelets	[31]
2010	Microfluor 96-well (ThermoLabsystems)	Collagen (2 μg/well)	2%	200,000/μL	Pre-labeled platelets with Calcein-AM	Plate reader without lysing platelets	[32]
2010	Immulon-2HB 96-well (Dynex Technologies)	Laminin-511 or Collagen (5 to 200 μg/mL)	3%	100,000/μL	Pre-labeled platelets with 4 μM Calcein-AM	Plate reader without lysing platelets	[33]
2010	Microfluor 96-well (ThermoLabsystems)	Fibrillar Collagen (1 μg/well)	2%	N/A	Pre-labeled platelets with 2 μM Calcein-AM, 30 min	Plate reader without lysing platelets	[34]
2011	Microfluor 2 high-affinity 96-well (Thermo Electron Co)	Fibronectin (1 μg/mL), Fibrinogen (100 μg/mL) or Collagen-I (2 μg/mL)	2%	200,000/μL	Pre-labeled platelets with 2 μM Calcein-AM at RT, 30 min	Plate reader without lysing platelets	[35]
2011	96-well (N/A)	Fibrinogen (concentration not mentioned)	0.35%	200/μL	Pre-labeled platelets with 7 μM Calcein-AM	Plate reader without lysing platelets	[36]
2011	Polystyrene 96-well (Nunc)	Fibrinogen (10 μg/mL)	N/A	200,000/μL	Pre-labeled platelets with 7 μM Calcein-AM at 37 °C, 30 min	Plate reader without lysing platelets	[37]
2012	Microfluor 96-well (ThermoLabsystems)	Collagen (1 μg/well)	2%	200,000/μL	Pre-labeled platelets with 2 μM Calcein-AM at RT, 30 min	Plate reader without lysing platelets	[38]
2012	96-well (N/A)	Fibrinogen (50 μg/mL)	1%	200,000/μL	Pre-labeled platelets with 2.5 μM Calcein-AM at RT, 15 min	Plate reader without lysing platelets	[39]
2013	96-well (N/A)	Collagen (40 μg/mL)	5%	N/A	Pre-labeled platelets with 2.5 μM Calcein-AM at RT, 15 min	Plate reader after lysing platelets	[40]
2013	Microfluor 96-well plates (ThermoLabsystems)	Collagen-I (20 μg/mL)	2%	200,000/μL	Pre-labeled platelets with 2 μM Calcein-AM at RT, 30 min	Plate reader without lysing platelets	[41]
2014	Tissue culture 96-well (Costa)	Fragment D98 (10 μg/mL)	1%	100,000/μL	Pre-labeled platelets with 10 μM Calcein-AM at 37 °C, 30 min	Plate reader without lysing platelets	[42]
2015	Microfluor 96-well (Thermo Labsystems)	Collagen (1 μg/well) or Fibrinogen (50 μg/well)	2%	200,000/μL	Pre-labeled platelets with 2 μM Calcein-AM at RT, 30 min	Plate reader without lysing platelets	[43]
2016	96-well (N/A)	Fibrinogen (50 μg/mL)	5%	500,000/well	Pre-labeled platelets with 7 μM Calcein-AM	Plate reader without lysing platelets	[44]
2016	96-well (Corning)	Collagen (100 μg/mL)	1%	N/A	Pre-labeled platelets with 10 ng/mL Calcein for 30min 37C	Plate reader without lysing platelets	[45]
2017	96-well (Greiner Bio-one, 655096)	Fibrinogen or Fibrinogen fragment D98 (10 μg/mL)	0.35%	200,000/μL	Pre-labeled platelets with 7 uM Calcein for 30min RT	Plate reader without lysing platelets	[46]
2020	96-well (N/A)	Collagen (20 μg/mL)	2%	200,000/μL	Pre-labeled platelets with 4 μM Calcein-AM at 37 °C, 60 min	Plate reader without lysing platelets	[47]

**Table 2:** Previous studies using BCECF-AM to measure platelet adhesion.

Year	Type of Plate	ECM Concentration	Blocking BSA	Platelet Concentration	Dye Concentration	Type of Measurement	Ref.
1996	96-well (N/A)	Collagen (100 μg/mL)	0.35%	300,000/μL	Pre-labeled platelets with 2 μM BCECF-AM at 37 °C, 30 min	Plate reader without lysing platelets	[48]
1997	96-well (Falcon)	Collagen-I or Fibrinogen (100 μg/mL)	0.0005%	375,000/μL	Pre-labeled platelets with 6 mM BCECF-AM at 37 °C, 30 min	Plate reader after lysing platelets	[49]
1997	96-well (Costar)	Fibronectin or Vitronectin (0.05 to 1.5 μg/well)	0.50%	300,000/μL	Pre-labeled platelets with 5 μM BCECF-AM at 37 °C, 40 min	Plate reader without lysing platelets	[50]
1997	96-well (Costar)	Fibronectin, Vitronectin, vWF, laminin (1 μg/well) or collagen-IV (5 μg/well)	0.50%	300,000/μL	Pre-labeled platelets with 5 μM BCECF-AM at 37 °C, 40 min	Plate reader without lysing platelets	[51]
1998	96-well (N/A)	Fibrinogen (0.01, 0.1 and 2 μg/well)	N/A	400,000/μL	Pre-labeled platelets with 12 μM BCECF-AM at 37 °C, 30 min	Plate reader without lysing platelets	[52]
1999	96-well (Immulon-2)	Fibrinogen (1 ng to 2 μg/well)	N/A	4,000,000/μL	Pre-labeled platelets with 6 μM BCECF-AM at 37 °C, 30 min	Plate reader without lysing platelets	[53]
1999	96-well (Costar)	Fibronectin, Vitronectin, vWF or Laminin (120 μg/mL)	0.50%	300,000/μL	Pre-labeled platelets with 5 μM BCECF-AM at 37 °C, 40 min	Plate reader without lysing platelets	[54]
2008	96-well (N/A)	Collagen or Fibrinogen (50 μg/mL)	1%	N/A	Pre-labeled platelets with BCECF-AM for 40 min (temperature and concentration N/A)	Microscopy	[55]
2010	96-well (N/A)	Collagen or Fibrinogen(50 μg/mL)	1%	N/A	Pre-labeled platelets with BCECF-AM for 30 min (temperature and concentration N/A)	Plate reader without lysing platelets	[56]
2018	96-well (N/A)	Collagen (10 μg/mL)	0.5% or 0.05%	N/A	Pos-labelling platelets with 12 μM BCECF-AM (incubation time and temperature N/A)	Plate reader after lysing platelets	[57]
2019	96-well (N/A)	Fibrinogen (100 μg/mL)	5%	200,000/μL	Pre-labeled platelets with BCECF-AM at 37 °C for 30 min (concentration N/A)	Plate reader after lysing platelets	[58]

Both BCECF-AM and Calcein-AM are hydrophobic acetoxymethyl ester (AM) derivatives and they are both excited (485 nm) and emit (530 nm) at identic wavelengths [59]. The addiction of AM that makes them membrane permeable [60]. BCECF-AM structure derives from carboxyfluorescein, with two extra carboxylate groups [60] and is sensitive to pH [61], while Calcein is a polyanionic fluorescein derivative that bears six negative charges and two positive charges at pH 7 [62] and is practically pH-insensitive [61].

Calcein-AM and BCECF-AM are nonfluorescent in their native form and can be loaded into the cell as the charge-neutral, membrane-soluble form. Once in the cytosol, these probes are modified by nonspecific endogenous esterases upon cleavage of ester groups [63,64]. The cleaved probe is fluorescent and negatively charged, and for this reason less permeable to the plasma membrane, thereby being retained inside the cell [64]. Multicharged molecules such as BCECF and Calcein have typically cell retention times greater than 2 h at 37 °C [64]. Generation of toxic photoproducts derived from BCECF, as well as products of the deesterification reaction, namely, acetate and formaldehyde, have been previously reported [65]. Evidences also point Calcein-AM as being toxic to and pumped out of several cell lines [62]. On the other hand, studies showed that BCECF is not toxic under certain conditions, as for example in vitro fertilized mouse eggs [66] or brainstem cells [67]. One can argue that the toxicity observed is dependent on the dye concentration and on the cell type studied.

Table 1 and Table 2 gather 13 and 25 studies performed over the years staining platelets with Calcein or BCECF, respectively. What is striking about these studies is the variability regarding the methodological settings. The tables highlight the following settings: type of plate; ECM protein, blocking agent (BSA) and dye concentrations; number of platelets and which type of measure was employed to detect the ECM-adherent platelets. Although not stated in all studies, the plates were acquired from varied companies. According to our observations, the ideal concentration of BSA for a successful inhibition of platelet adhesion depends on plate type used. The BSA titration studies revealed that all concentrations between 0.00075% and 4% BSA significantly reduced platelet adhesion to Invitrogen™plates. Surprisingly, the same range of BSA concentrations failed to inhibit platelet adhesion to the surface of Greiner Bio-One (781186) one or Falcon (353072) plates, which were some of the plate brands used in studies ofTable 1 and Table 2. This could lead to a non-specific platelet adhesion.

The appropriate platelet concentration was also investigated, considering the feasibility of the test and the number of adherent platelets. A minimum of 4 × 10^4^ platelets/µL should be used, since no statistical difference was observed when a lower concentration of 2 × 10^4^ platelets/µL was used. A titration assay ranging from 4 × 10^4^ to 1.6 × 10^6^ platelets/µL showed platelet adhesion linearity with a regression of 0.9971 and a concentration of 8 × 10^4^ platelets/µL was considered ideal for a reproducible assay, since it had a lower standard deviation, compared to higher concentrations, maintaining a measurable fluorescence signal. Furthermore, this number of platelets requires less blood, which also increases the feasibility of the assay for high-throughput screening.

Different ECM proteins constitute substrates for platelets adhesion, being collagen, fibrinogen, fibronectin, vitronectin, vWF, and laminin some of the proteins used in previous adhesion studies (Table 1 and Table 2). In our experiment, we tested a panel of different ECM protein coatings, and titrated the best concentration for that particular protein, by platelet staining with BCECF. Interestingly, substrates such as fibrinogen presented the highest number of adherent platelets, reaching the maximal adhesion at 2 mg/mL, being the plasmatic concentration of this protein between 2–4 mg/mL [68], which is an important factor for platelet activation. The same seems to happen in the context of adhesion. However, most of the previous assays employed fibrinogen in a concentration ranging 50–100 µg/mL, which might not be the concentration leading to the maximal platelet adhesion. This supports the need of a standardized protocol to measure platelet adhesion. On the other hand, the least adhesive substrate seems to be vitronectin, which failed to induce significant platelet adhesion in our study. The adhesion to collagen type III did not reach statistical differences, although an increased adhesion tendency could be observed. Regarding one of the most commonly used protein to study platelets adhesion, collagen-I, in low concentrations, as used in some of the studies, failed to trigger platelet adhesion in a statistically significant manner, which was only reached with concentrations of 2–8 µg/mL or 64 µg/mL for non-fibrilar or fibrilar collagen I, respectively. Interestingly, laminins-411 and -511 at concentrations ranging 7.5–15 µg/mL, and 10 and 15 µg/mL, respectively, are also more potent substrates for platelet adhesion, compared to other tested substrates. Altogether, the discrepancies in the concentration range of ECM protein coatings between studies reinforce the need for standardized protocols, as here proposed, which contributes for the reproducibility of results. Most importantly, our experiments in which adherent platelets to collagen-I were stained with either BCECF-AM or Calcein-AM, highlighted that BECF-AM, although it presented higher standard deviation values, did reach statically significant differences. Using BCECF-AM, the number of adherent platelets detected was much higher than when platelets were pre-stained with Calcein-AM. Calcein-AM staining of platelets failed to reach significant detectable differences between platelets adhesion to collagen-I coated or non-coated surfaces.

Regarding the dye fluorescence detection method employed can be either by analyzing the cells under a fluorescence microscope or by quantitatively measuring the fluorescence, using a fluorescence plate reader. An advantage of this assay is that both analysis can be performed. The fluorescence measure using a plate reader assures that the quantitative analysis of the fluorescence-based assay is reliable. Furthermore, our studies demonstrate that microscopic analysis can be posteriorly performed, allowing the analysis not only the fluorescence but also the cell morphology, and understand if platelets spread more or less in a given substrate. Importantly, in order to avoid photo bleaching, microscopy should be performed after the fluorescence quantitative measurement in the plate reader. To further test the method’s sensitivity, a collagen-I adhesion inhibitor, which blocks the integrin α2β1, successfully decreased the adhesion of platelets, which was detected by BCECF-AM staining.

Lastly, and in order to validate the assay quality, the Z′-factor was calculated. An optimal Z′-factor value (higher than 0.5) was attained employing the following experimental settings: Plate coated with 1 mg/mL of fibrinogen, as it led to maximal platelet adhesion; blocking agent BSA in a concentration of 0.03%; platelets in a concentration of 8 × 10^4^/µL in 10 µL incubated for 1 h at 37 °C, and stained with 4 µg/mL BCECF-AM in 20 µL for 30 min at 37 °C. This analysis is important, as it states the robustness of the assay, which might contribute for a more reproducible assay. This is an essential aspect, as reproducibility is a major problem in science. In addition, the Z-Factor value assures the compatibility of the assay with a high-throughput screening format. 

The present assay is performed in a 2D setting; however, in an updated version of the assay, 3D matrix gels could be potentially applied to assess platelet adhesion under near-physiological conditions. The current available technology does not allow the evaluation of platelet adhesion under shear stress conditions in a high-throughput screening format, which constitutes a limitation to the study. Therefore, this assay cannot be applied to study shear-stress dependent receptors, such as GPIb and its interaction with vWF.

## 4. Materials and Methods

### 4.1. Materials

The following microplates were used in this study: Nunc MaxiSorp™ 96-well clear flat-bottom (44-2404-21, Invitrogen™, Carlsbad, CA, USA), Falcon^®^ 96-well clear flat-bottom tissue culture (TC)-treated (Corning, 353072, Corning, NY, USA), Greiner Bio-One 96-well clear flat-bottom (Greiner Bio-One, 655180, Kremsmünster, Austria) and Greiner Bio-One 384-well clear flat-bottom (Greiner Bio-One, 781186). Prostaglandin E1 (Sigma, P7527, St. Louis, MO, USA) was used for washed platelet preparation. Microplate wells were coated with the following ECM proteins: Fibrinogen (Sigma, F8630), fibronectin (Sigma, F4759), non-fibrillar collagen type-I (Sigma, C7661), fibrillar collagen type-I (möLab, 0203009, Langenfeld, Germany), collagen type-III (Sigma, C4407), collagen type-IV (Sigma, C7521), laminin-411 (BioLamina, LN411-02, Sundbyberg, Sweden), laminin-511 (BioLamina, LN511-02), and vitronectin (Sigma, 5051). Collagen-related peptide (CRP), also used for coating, was purchased from University of Cambridge. BSA (Sigma, A7906) was used to block the non-specific binding to the wells. The following dyes were used to detect platelet adhesion: BCECF-AM (Sigma, B8806), Calcein-AM (Sigma, 17783) and Sudan Black B (Sigma, 199664). TC-I 15, an α2β1 integrin inhibitor was obtained from R&D Systems (4527, Minneapolis, MN, USA). The following equipment was used in this study: Plate reader (PerkinElmer, Victor X3) and microscope (Nikon Eclipse Ti2).

### 4.2. Washed Platelet Preparation

Washed platelets were prepared as previously described [57]. Human blood was collected from healthy volunteers, who had not been medicated in the previous 10 days, using a 10 mL tube (SARSTEDT, S-Monovette, Nümbrecht, Germany), maintaining a 6:1 ratio of whole blood to ACD (117 mM sodium citrate, 78 mM citric acid and 282 mM dextrose). Platelet-rich plasma (PRP) was obtained by two centrifugation steps at 500× *g* for 15 min at room temperature. Aliquots of PRP (1 mL) were distributed in 2 mL centrifuge tubes containing 22.5 μM prostaglandin E1 (PGE1) and centrifuged at 10,000× *g* for 30 s. The platelet pellet was resuspended in magnesium- and calcium-free tyrode buffer at pH 6.2 (17 μM PGE1, 137 mM NaCl, 2.7 mM KCl, 3 mM NaH_2_PO_4_, 10 mM hepes, 1.25 mM NaHCO_3_, and 5.6 mM dextrose), and this procedure was repeated once more. Finally, platelets were resuspended in tyrode buffer, pH 7.4, in absence of PGE1 but containing 2 mM CaCl_2_ and 1 mM MgCl_2_. All participants provided informed consent in accordance with the Helsinki Declaration and all protocols were approved by the Swiss ethics committee (project-ID 2017–00732, date of approval 23.05.2017, Commission cantonale d’éthique de la recherche sur l’être humain).

### 4.3. Study Design

#### 4.3.1. 96-Well Plate and BCECF-AM

**Figure 7:**
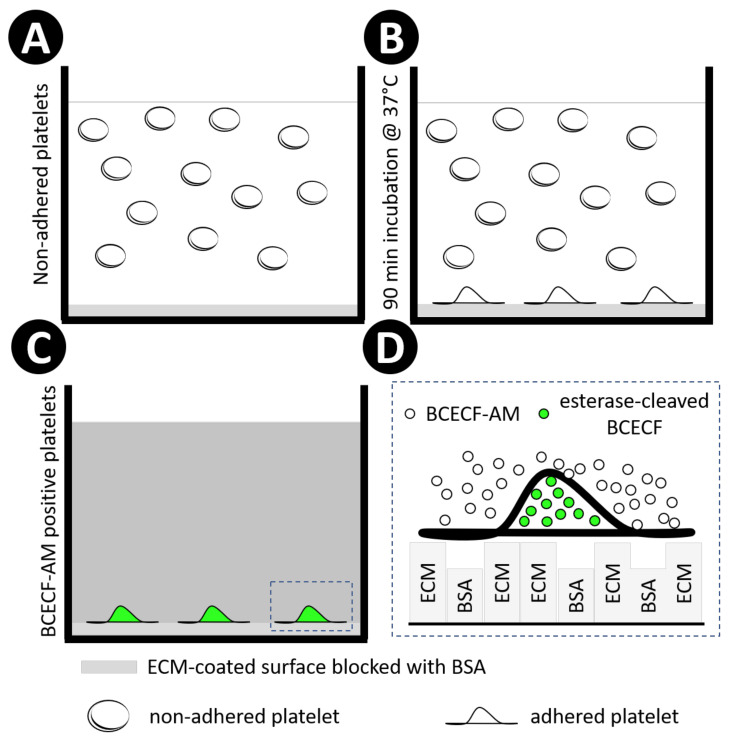
Schematic representation of the study design using BCECF-AM. Extracellular matrix proteins were employed to coat 96- or 384-well microplates, followed by BSA blocking, both incubated for 1 h at 37 °C. Next, human washed platelets were added into wells (**A**). and incubated for 90 min at 37 °C (**B**). Non-adherent platelets were washed away with tyrode buffer, and adherent platelets were incubated with diluted BCECF-AM for 30 min at 37 °C. The excess of BCECF-AM was removed by washing the plate 3 times with tyrode buffer (**C**). Non-fluorescent BCECF-AM is permeable to the platelet membrane. Once inside the platelet, intracellular esterases cleave the ester bond, releasing BCECF, which is the fluorescent form of the molecule. In addition, the cleavage of lipophilic blocking groups by esterases, leads to a charged form of BCECF, which leaks out of cells more slowly than BCECF-AM [69]. Rectangular boxes represent ECM and BSA coating(**D**).

ECM proteins were added to a 96-well plate in different concentrations (50 μL/well) followed by incubation for 1 h at 37 °C. The plate content was discarded, and the wells were washed 3 times with distillated water (100 μL/well). Next, different concentrations of BSA were added to the well to block non-specific binding of platelets. After 1 h incubation at 37 °C, the wells coated with ECM proteins and blocked with BSA were washed 3 times with distilled water (100 μL/well). Immediately after, 50 μL of different concentration of platelets were added and incubated for 1 h at 37 °C. Non-bond platelets were washed with tyrode buffer, pH 7.4 (100 μL/well), and adherent platelets were stained with different concentrations of BCECF-AM diluted in tyrode buffer, pH 7.4 (50 μL/well), for 30 min at 37 °C. Next, the BCECF-AM excess was removed by washing the plate 3 times with tyrode buffer, pH 7.4 (100 μL/well). Fluorescent signal of BCECF-AM was measured using a plate reader (VictorX, PerkinElmer) with the parameters shown in Table 3. Fluorescent signal of BCECF-AM was also measured after lysing adhered platelets. For that, lysis buffer (0.1% SDS in 30 mM Tris pH 8.8) was added after washing the excess of BCECF-AM. For microscopy purposes, images from non-lysed platelets were acquired using a fluorescence microscope. Figure 7 schematically represents the study design using BCECF-AM.

#### 4.3.2. 96-Well Plate and Calcein-AM

For the experiment using Calcein-AM, platelets were pre-stained following the protocol previous published by several authors (see Table 1). Briefly, PRP obtained by centrifugation of the whole blood was incubated with Calcein-AM (2 µg/mL) for 1 h at 37 °C. Next, washed stained-platelets were obtained after a series of washing steps as described in the Section 4.2. Adhesion of pre-stained platelets was measured as the platelets stained with BCECF-AM in a 96-well plate. Fluorescence signal of Calcein-AM was measured using a plate reader with the parameters shown in Table 3. Fluorescent signal of Calcein-AM was also measured after lysing adherent platelets. For that, lysis buffer (0.1% SDS in 30 mM Tris pH 8.8) was added to adherent platelets.

#### 4.3.3. 384-Well Plate and BCECF-AM

Fibrinogen was added to a 384-well plate in different concentrations (0.03 to 8 mg/mL—20 μL/well), followed by incubation for 1 h at 37 °C. The content of the plate was discarded, and the wells were washed 3 times with distilled water (20 μL/well). Next, BSA 0.03% was added to the wells to block non-specific binding of platelets. After 1 h incubation at 37 °C, the wells coated with fibrinogen and blocked with BSA were washed 3 times with distilled water (20 μL/well). Immediately after, platelets (8 × 10^4^/µL in 10 µL) were added and incubated for 1 h at 37 °C. Non-adherent platelets were washed away with tyrode buffer, pH 7.4 (20 μL/well), and adhered platelets were incubated with BCECF-AM diluted in tyrode buffer, pH 7.4 (4 µg/mL, 20 μL/well), for 30 min at 37 °C. Next, the excess of BCECF-AM was washed 3 times with tyrode buffer, pH 7.4 (20 μL/well). BCECF-AM fluorescence signal was measured using a plate reader with the parameters as shown in Table 3. For microscopy purposes, images from non-lysed platelets were acquired using a fluorescent microscope.

### 4.4. Data Analysis

To validate the assay, fibrinogen (1 mg/mL) was added to half of the 384-well microplate (176 wells—right side). In the other half, water was added (176 wells—left side). As a control, platelet adhesion on plastic was measured in the first and last columns. The following formula was used to calculate the Z′-factor value, as described in a previous publication [22].

(1) $$Z^{\prime }\mathrm{factor}=1-\frac{3\mathrm{SD}\;\mathrm{of}\;\max\;\mathrm{response}+3\mathrm{SD}\;\mathrm{of}\;\min\;\mathrm{response}}{\mathrm{mean}\;\mathrm{of}\;\max\;\mathrm{response}-\mathrm{mean}\;\mathrm{of}\;\min\;\mathrm{response}}$$

Unless otherwise stated, triplicates were performed in every experiment using platelets from four different donors. The data are expressed as mean ± standard error of mean (SEM). Each measurement represents the average of two wells within the same condition. Statistical differences among groups were analyzed by one-way analysis of variance (ANOVA) followed by Dunnett’s test. T-test was used to determine whether there was a difference in collagen-coated and non-coated wells for assay linearity of platelet adhesion in a 96-well plate. Two-way ANOVA followed by Tukey’s post hoc test was used to determine the difference between BCECF-AM and Calcein-AM techniques. Probability value (*p*) < 0.05 was considered statistically significant. GraphPad Prism 6.0 (San Diego, CA, USA) was used to perform the statistical analysis.

## 5. Conclusions

Our studies importantly highlight the potential advantages and disadvantages of different methodological approaches to detect platelet adhesion by measuring fluorescent dyes. As an essential step of the thrombus formation process, platelet adhesion can be studied in vitro. An advantage of the proposed in vitro assay is its versatility, as different ECM proteins representative of the ECM of the healthy or injured vessel can be studied, and their individual effect on platelet adhesion pin-pointed. Furthermore, drug discovery to identify new adhesion inhibitors can also be performed, and murine, human platelets or platelets from patients with platelet-related pathologies can be studied for their adhesive capacities. Lastly, the development of a high-throughput assay, which is both fast and inexpensive, is of extreme interest for drug development. The standardization of a methodological approach that can fulfill all these criteria, and that can be employed by both researchers, drug developers and clinicians, to analyze platelet adhesion in a reproducible and comparable way, is essential. In the present work, we optimized and standardized a high-throughput method able to measure quantitatively platelet adhesion to different substrates, allowing also morphological characterization of the adherent platelets. The methodological approach is detailed in the standard operating procedure (SOP) present in the supplemental material.

## Figures and Tables

**Table 3:** Plate reader parameters for BCECF-AM and Calcein-AM.

Dye	BCECF-AM and Calcein-AM
Signal	Fluorescence
Excitation:	485 nm
Emission:	535 nm
Measurement time:	0.1 s
Lamp energy (CW):	15000
Emission side:	Above
Temperature:	22 °C

## Supplementary Materials

Supplementary Materials can be found at https://www.mdpi.com/1422-0067/21/18/6539/s1.

## Abbreviations

AM	Acetoxymethyl
BCECF	2′,7′-Bis-(2-Carboxyethyl)-5-(and-6)-Carboxyfluorescein
BSA	Bovine Serum Albumin
CRP	Collagen-Related Peptide
ECM	Extracellular Matrix
Fbg	Fibrinogen
N/A	Not Available
*p*	Probability value
PGE1	Prostaglandin E1
RT	Room Temperature
PRP	Platelet-Rich Plasma
PPP	Platelet-Poor Plasma
SEM	Standard Error of the Mean
SBB	Sudan Black B
SD	Standard Deviation
SOP	Standard Operating Procedure
FI	Fluorescence Intensity
