# Supplementary material for: Standardization and Validation of Fluorescence-Based Quantitative Assay to Study Human Platelet Adhesion to Extracellular-Matrix in a 384-Well Plate
Source: Int J Mol Sci. 2020 Sep 7;21(18):6539. doi: 10.3390/ijms21186539 (PMC7554887; doi:10.3390/ijms21186539)
Supplement: Supplementary file 1 [file ijms-21-06539-s001.pdf]

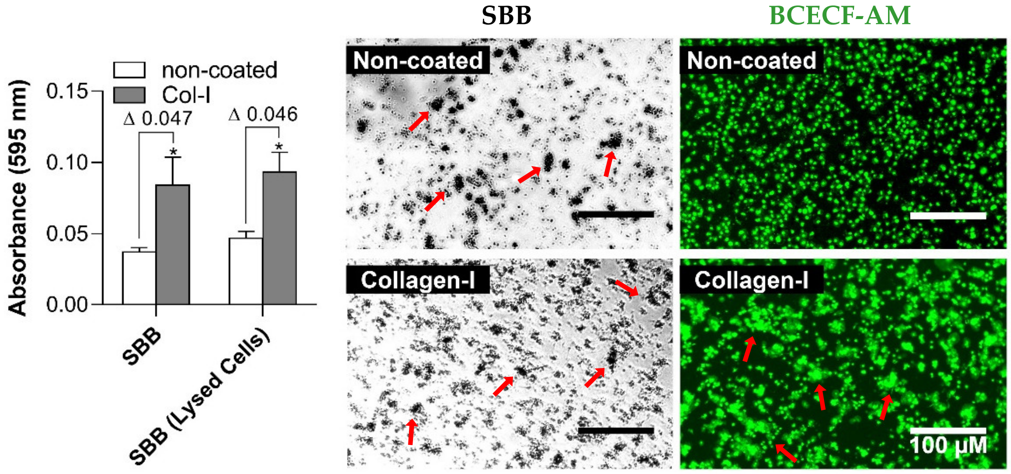

Supplemental Figure 1. Platelet adhesion detection with SBB. Coating of 96-well microplates with collagen-I (4 μg/mL in 50 μL), or incubation with distillated water, were performed for 1 h at 37°C. After blocking the wells with BSA (0.03%), human washed platelets (8x10^4 /μL in 100μL) were added, followed by incubation for 1 h at 37°C. Non-adherent platelets were washed away with tyrode buffer. Sudan Black B (SBB) was used as previous published by Xu and cols [1]. Briefly, aliquots of PRP (1 mL) were distributed in 2 mL centrifuges tubes containing 22.5 μM prostaglandin E1 (PGE1) and centrifuged at 10 000 xg for 30 sec. The platelet pellet was resuspended in magnesium- and calcium-free tyrode buffer pH 6.2, and platelets were incubated for 30 min with a filtered solution of SBB in 70% ethanol, added at a volume ratio of 1:20. Next, platelets were washed in presence of prostaglandin E1 and resuspended in tyrode buffer as described in the method section 4.2. Absorbance intensity was measured using a plate reader (VictorX, PerkinElmer). Images were acquired using a fluorescence microscopy (Eclipse Ti2, Nikon) with a 20x objective and SBB-stained platelets were compared to BCECF-AM-labeled platelets. Red arrows indicate platelet aggregates. Absorbance values of collagen I-coated surfaces blocked with BSA was compared to BSA alone (non-coated) for each experimental group by test-t (*$P < 0.05$, values presented as SEM resulting from duplicate average of four independent experiments).

Supplemental Standard Operating Procedures (SOP): Human Platelet Adhesion Assay

I. Material

I.1. Plates references

Nunc MaxiSorp™ 96-well clear flat bottom (Invitrogen™, 44-2404-21)	Greiner Bio-One 384-well clear flat bottom (Greiner Bio-One, 781186)

I.2. ECM proteins references

Fibrinogen (Sigma, F8630), fibronectin (Sigma, F4759), non-fibrillar collagen type-(Sigma,C7661), fibrillar collagen type-1 (möLab, 0203009), collagen type-IV (Sigma, C7521), laminin-411 (BioLamina, LN411-02), laminin-511 (BioLamina, LN511-02), and collagen-related peptide (CambCol Laboratories, CRP-XL).

I.3. Reagents References

- BSA (Sigma, A7906).
- Tyrode Buffer containing 137mM NaCl (S7653, Sigma), 2.7mM KCl (P9333, Sigma), 3mM NaH_2PO_4 (S3139, Sigma), 10mM Hepes (A1069, ITW Reagents), 5.6mM Glucose (G5400, Sigma), 10mM $NaHCO_3$ (C2680, Bachmann-Lehrmittel AG), 1mM Magnesium Chloride Hexahydrate (63068, Sigma), and 2 mM Sodium Chloride (C1016, Sigma).

I.4. Dye Reference

- BCECF-AM (Sigma, B8806)

I.5. Devices References

- Plate reader (PerkinElmer, Victor X3)
- Microscope (Nikon Eclipse Ti2)

II. Experimental Protocol

1. Add each substrate solution diluted in distillate water (dH_2O) into the wells:
    a. 20 µL for 384 -well plate
    b. 50 µL for 96-well plate
    Note: Submaximal concentrations of each substrate
    Fibrinogen (1 to 2 mg/mL), Fibronectin (20 µg/mL), Non-fibrillar collagen type-(2 to 4 µg/mL), Fibrillar collagen type-1 (64 µg/mL), Collagen IV (2 to 4 µg/mL), Laminin 411 (7.5 to 8.75 µg/mL), Laminin 511 (10 to 15 µg/mL), and Collagen-related peptide (10 µg/mL).
2. Spin down the plate containing the ECM using a centrifuge. Note: speed suggestion 300 rpm for 60 seconds.
3. Incubate the plate at 37°C for 1 hour.
4. Discard the unbound ECM proteins in a single movement by turning the plate upside down followed by a sudden vertical motion over a sink.
5. Wash the wells with:
    a. 30 µL of dH_2O for 384-well plate.
    b. 100 µL of dH_2O for 96-well plate.
6. Discard the dH_2O in a single movement by turning the plate upside down followed by a sudden vertical motion over a sink.
7. Wash the wells with:
    a. 30 µL of dH_2O for 384-well plate
    b. 100 µL of dH_2O for 96-well plate.
8. Discard the dH_2O in a single movement by turning the plate upside down followed by a sudden vertical motion over a sink. Wash the wells with:
    a. 30 µL of dH_2O for 384-well plate
    b. 100 µL of dH_2O for 96-well plate.
9. Discard the dH_2O in a single movement by turning the plate upside down followed by a sudden vertical motion over a sink. Block the wells using 0.03% BSA:

    a. 30 µL of BSA for 384-well plate
    b. 100 µL of BSA for 96-well plate.
10. Spin down the plate containing the ECM proteins using a centrifuge – Suggestion: 300 rpm – 60 seconds.
11. Incubate the plate at 37°C for 1 hour.
12. Discard the excess of BSA in a single movement by turning the plate upside down followed by a sudden vertical motion over a sink.
13. Wash the wells with:
    a. 30 µL of dH_2O for 384-well plate
    b. 100 µL of dH_2O for 96-well plate.
14. Discard the dH_2O in a single movement by turning the plate upside down followed by a sudden vertical motion over a sink. Wash the wells with:
    a. 30 µL of dH_2O for 384-well plate
    b. 100 µL of dH_2O for 96-well plate
15. Discard the dH_2O in a single movement by turning the plate upside down followed by a sudden vertical motion over a sink. Wash the wells with:
    a. 30 µL of dH_2O for 384-well plate
    b. 100 µL of dH_2O for 96-well plate
16. Discard the dH_2O in a single movement by turning the plate upside down followed by a sudden vertical motion over a sink. Note: Keep dH_2O in the wells in case platelets are not ready to be added, avoid ECM-coated wells getting dry.
17. Add human washed platelets into the wells under different experimental conditions at a concentration of 80 000 / µL diluted in tyrode buffer pH 7.4.
18. Spin down the plate containing the ECM using a centrifuge – Suggestion: 300 rpm – 60 seconds.
19. Incubate the plate for 1 hour at 37°C to allow platelets to adhere.
20. Discard the non-adherent platelets in a single movement by turning the plate upside down followed by a sudden vertical motion over a sink.
21. Wash the wells with:
    a. 30 µL of tyrode buffer for 384-well plate
    b. 100 µL of tyrode buffer for 96-well plate.
22. Discard the tyrode buffer in a single movement by turning the plate upside down followed by a sudden vertical motion over a sink.
23. Add the BCECF-AM solution at the concentration of 4 µg/mL diluted in tyrode buffer:
    a. 20 µL for the 384 well-plate
    b. 50 µL for the 96-well-plate.

    Note: BCECF-AM is light sensitive and it has to be protected from light.
24. Spin down the plate containing the ECM proteins using a centrifuge – Suggestion: 300 rpm – 60 seconds.
25. Incubate the plate for 30 minutes at 37°C.
26. Discard the excess of BCECF-AM in a single movement by turning the plate upside down followed by a sudden vertical motion over a sink.
27. Wash the wells with:

a. 30 μL of tyrode buffer for 384-well plate
b. 100 μL of tyrode buffer for 96-well plate.

28. Discard the tyrode buffer in a single movement by turning the plate upside down followed by a sudden vertical motion over a sink. Add tyrode buffer to the wells:
    a. 30 μL of tyrode buffer for 384-well plate
    b. 100 μL of tyrode buffer for 96-well plate.
29. Record fluorescence with the microplate reader spectrophotometer with 485nm as excitation wavelength and 535nm as emission wavelengths.
30. Images can be acquired using a fluorescence microscope with a 20x objective (Nikon CFI S Plan Fluor ELWD) and a DS-Ri2 camera (4908×3264 pixel).
31. At the end of the experiment discard the plates in a proper waste bin.

Reference

1. Xu, X.-X.; Gao, X.-H.; Pan, R.; Lu, D.; Dai, Y. A simple adhesion assay for studying interactions between platelets and endothelial cells in vitro. Cytotechnology 2010, 62, 17–22, doi:10.1007/s10616-010-9256-2.
